# Supplementary material for: Small RNAs regulation and genomic harmony: insights into allopolyploid evolution in marsh orchids (Dactylorhiza)
Source: New Phytol. 2026 Feb 5;250(1):648–60. doi: 10.1111/nph.70966 (PMC12961254; doi:10.1111/nph.70966)

***New Phytologist* Supporting Information**

Article title: **Small RNAs regulation and genomic harmony: insights into allopolyploid evolution in marsh orchids (*Dactylorhiza*)**

Authors: Mimmi C. Eriksson, Matthew Thornton, Emiliano Trucchi, Thomas M. Wolfe, Francisco Balao, Mikael Hedrén, Ovidiu Paun

Article acceptance date: 15 December 2025

The following Supporting Information is available for this article:

Tables S1-S9 Available separately as xlxs tables.

Fig. S1 Distribution of normalised reads counts for different smRNA lengths over genomic regions (promoter/1000 bp upstream, exons, introns and intergenic) in the four tested species. For each plot, the central, coloured horizontal line indicates the median, solid whiskers represent the 0.25–0.75 quantile range, and dashed whiskers extend to the 0.05 and 0.95 quantiles. fuc = *D. fuchsii*, inc = *D. incarnata*, maj = *D. majalis*, tra = *D. traunsteineri*.

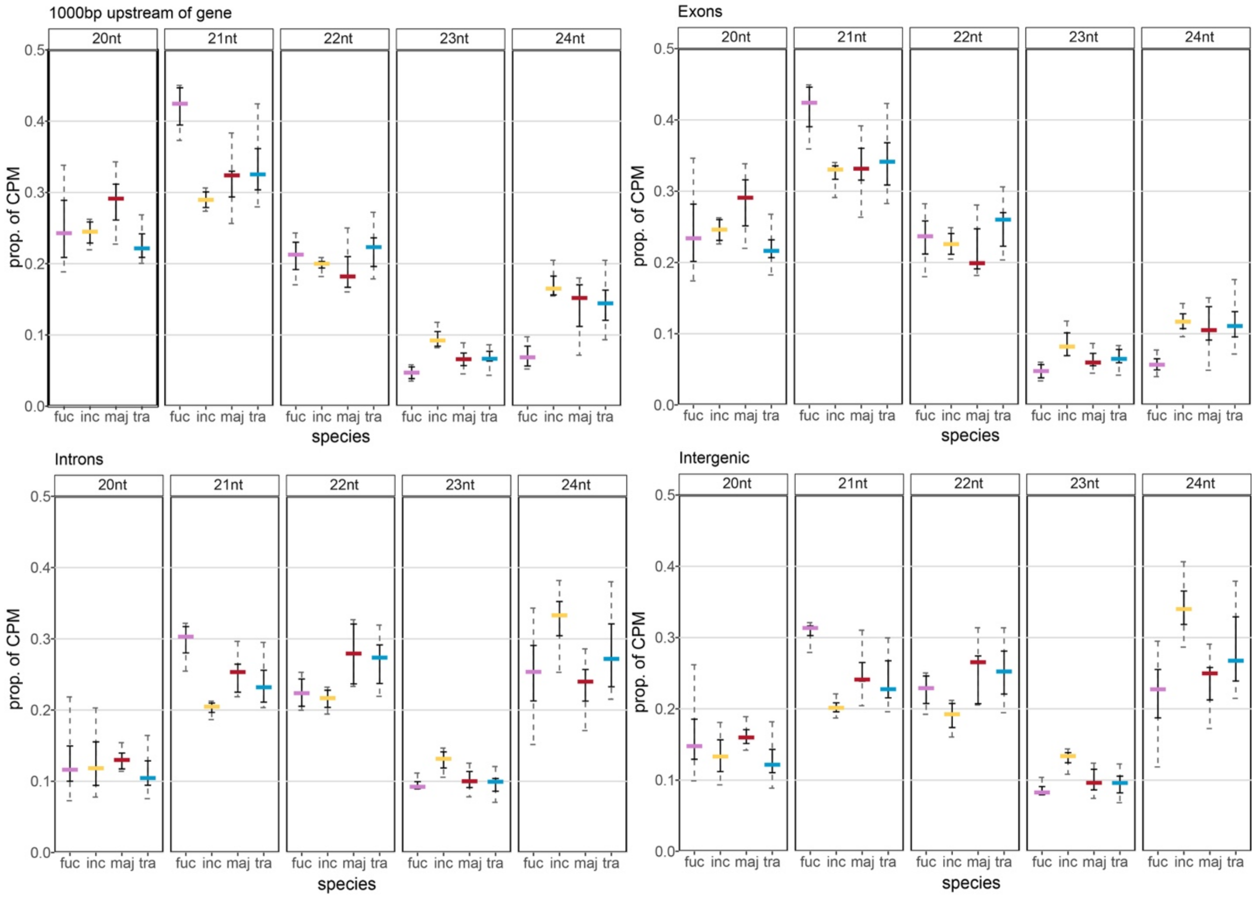

Fig. S2 Number of DT regions (i.e., peaks) in genes and TEs for each comparison between a polyploid (4x) and a diploid (2x) for all three data sets, 20-24 nt 20-23 nt and 24 nt smRNAs. The top panel contains the number of target regions where the polyploid is over regulated compared to the diploid, whereas the bottom panel contains the number of target regions where the diploid is over targeted. The coloured boxed below the figure represents the comparison shown in the corresponding column.

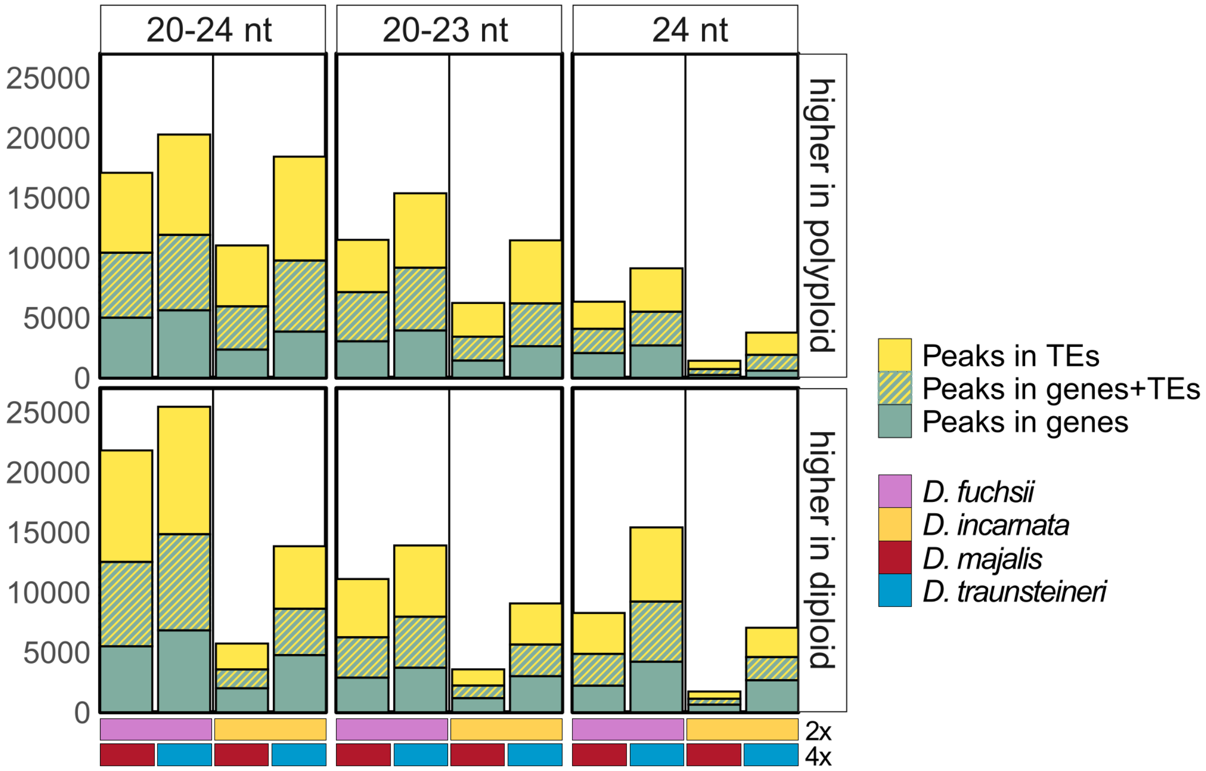

Fig. S3 Number of DT regions in all datasets 20−24 nt, 20−23 nt and 24 nt smRNAs for each genomic interaction (additive, transgressing or dominant to either diploid). The top row contains DT regions in genes, the middle row shows DT regions in TEs and the bottom row shows target regions found outside annotated genic and TE regions, e.g. intergenic. Colours represent, red - *D. majalis*, blue - *D. traunsteineri* and striped pattern - number of DT regions showing the same genomic interaction in both *D. majalis* and *D. traunsteineri*. The two annotation columns along the Y−axis represent the type (coloured bars according to legend) of genomic interaction Fig. 4, while dots connected with lines show the direction. Letters above each column of dots represent **F** - *D. fuchsii*, **P** - polyploid and **I** - *D. incarnata*.

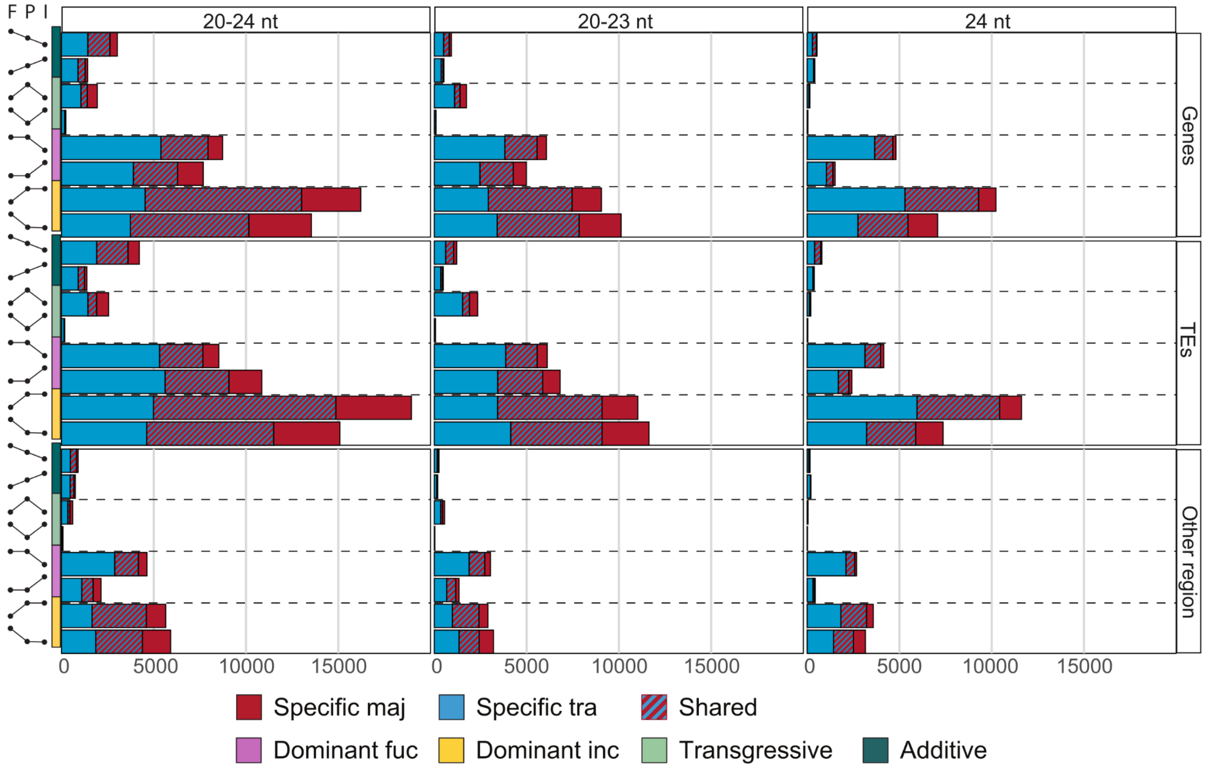

Fig. S4 Patterns of genomic interactions comparing logFC values for *D. majalis* (left) and *D. traunsteineri* (right) towards either diploid, *D. fuchsii* on the Y-axis and *D. incarnata* on the X-axis for all target regions (i.e., 20−24 nt smRNAs) found in annotated genes. (a-b) shows with coloured symbols transgressive and additive patterns; whereas (c-d) is dominant to either diploid, according to the legend.

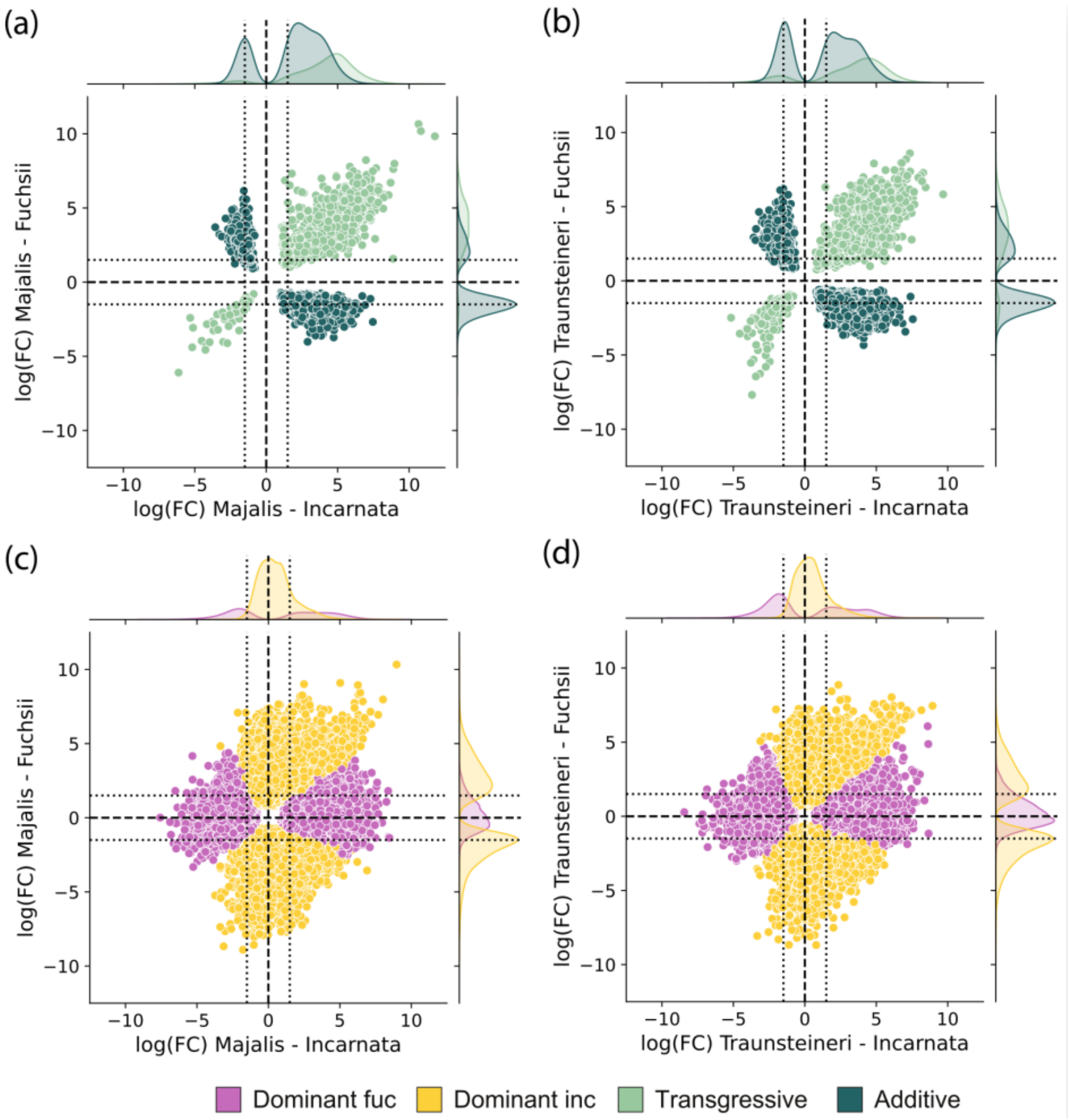

Fig. S5 Proportions of target regions within each genomic interaction (additive, transgressing or dominant to either diploid) for each genomic region (1000 bp upstream/promoter, exon, intron). Colours are according to legend. Letters in legend represent **F** - *D. fuchsii*, **p** - polyploid and **I** - *D. incarnata* while symbols represent level of smRNA between the diploids and polyploid.

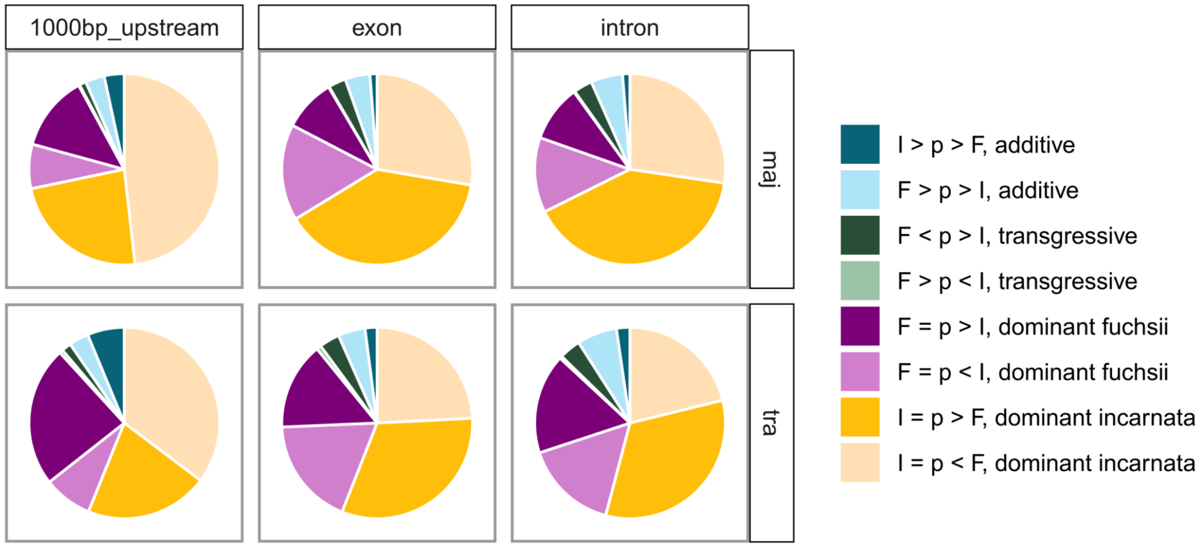

Fig. S6 MA plots showing differences between *D. majalis* and *D. traunsteineri* for TEs types grouped together, the central dashed line marks 0 while the dotted lines mark logFC -1.5 and 1.5. Negative logFC values represent over-targeting in *D. majalis* as compared to *D. traunsteineri*, whereas positive logFC values indicate over-targeting in *D. traunsteineri*. (a) and (b) show 20−23 nt smRNAs while (c) and (d) show 24 nt smRNAs. (a) and (c) show DT regions specific for each smRNA group, i.e. 20−23 nt or 24 nt, while (b) and (d) show DT regions for both 20−23 nt and 24 nt smRNAs.

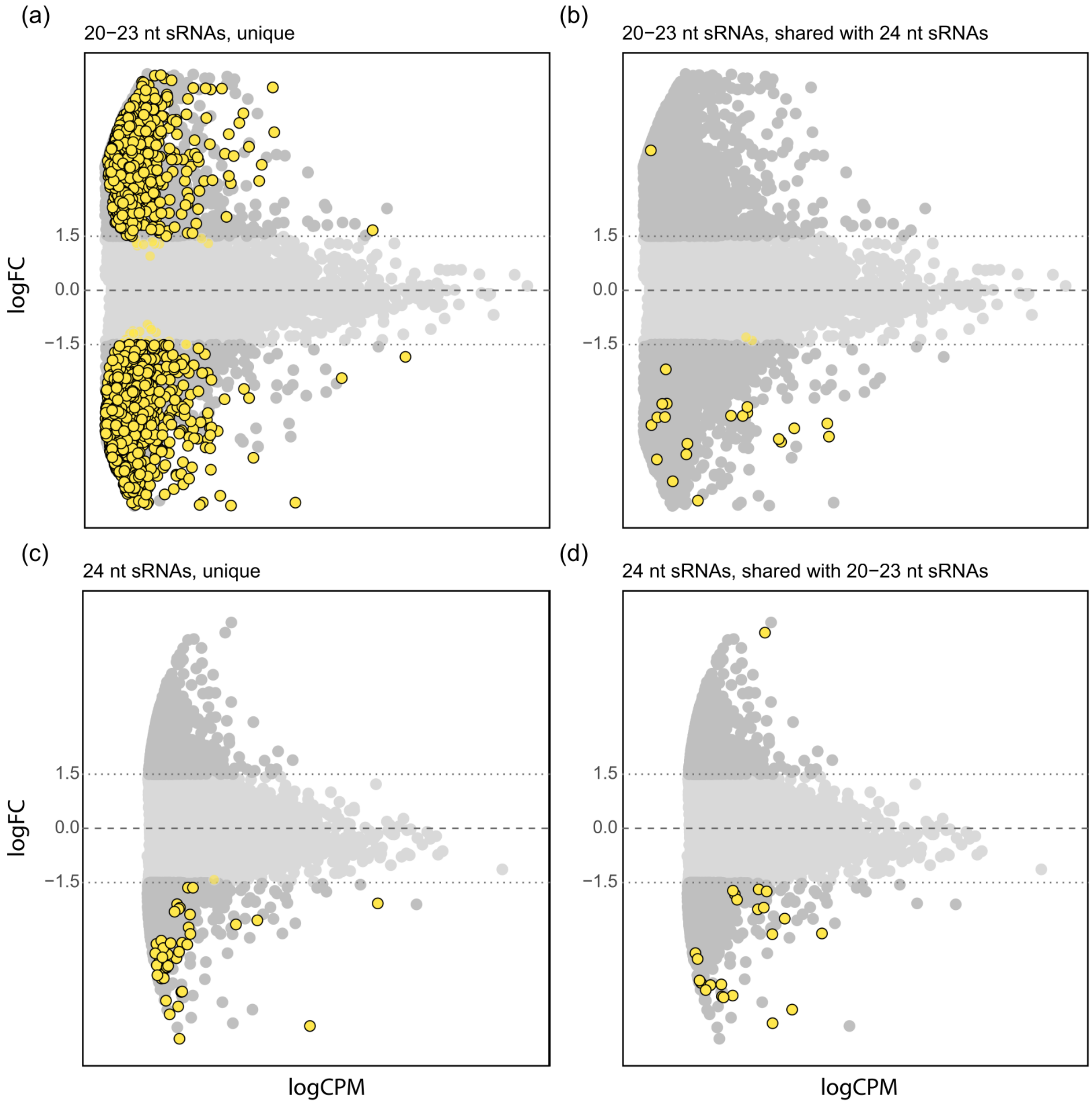

Fig. S7 MA plots showing differences between *D. majalis* and *D. traunsteineri* for TEs, the central dashed line marks 0 while the dotted lines mark logFC -1.5 and 1.5. Negative logFC values represent over-targeting in *D. majalis* as compared to *D. traunsteineri*, whereas positive logFC values indicate over-targeting in *D. traunsteineri*. Colours correspond to different types of TEs, indicated by column headers. Rows correspond to smRNAs, where the 1st and 2nd row show 20−23 nt smRNAs and 3rd and 4th show 24 nt smRNAs. The 2nd and 3rd row show target regions (i.e., peaks) that are DT for both 20−23 nt and 24 nt smRNAs.

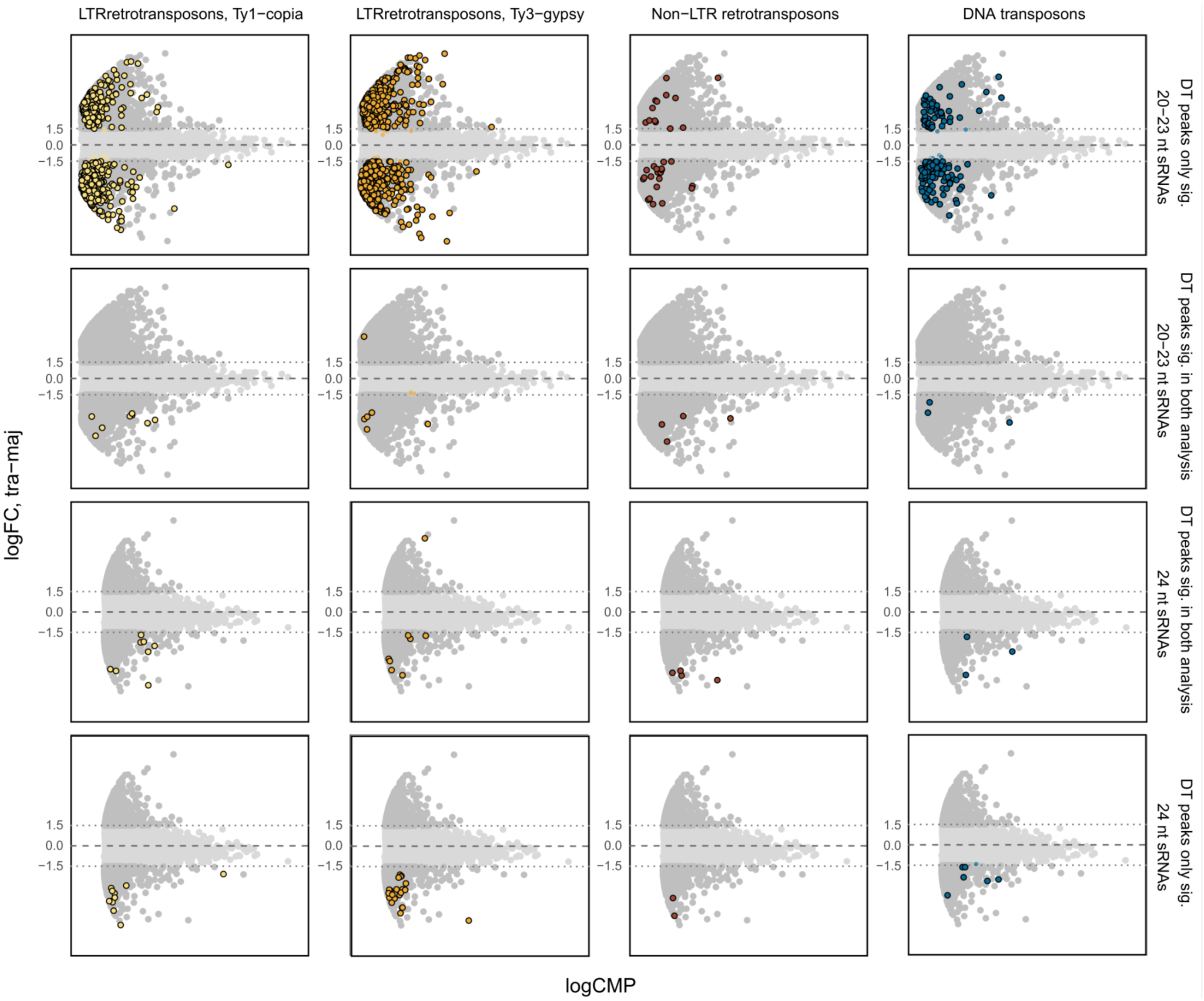

Fig. S8 Count of DT regions between *D. majalis* and *D. traunsteineri*, the coloured points of the plots in the 1st row (i.e. 20−23 nt smRNAs) in Fig. S7. Here Ty3-gypsy has been divided into the next annotation level.

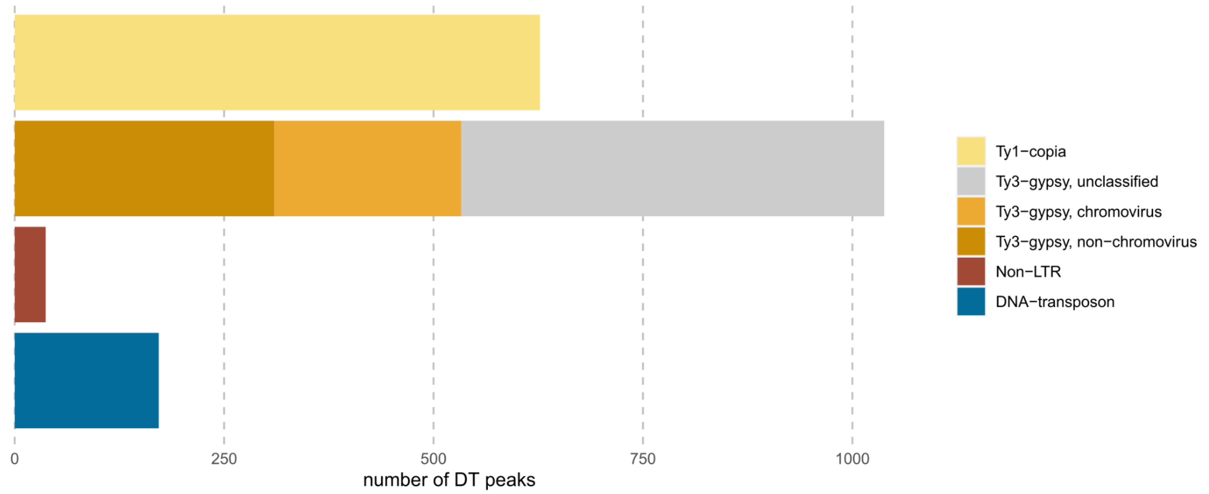

Fig. S9 Patterns of genomic interactions comparing logFC values for *D. majalis* (left) and *D. traunsteineri* (right) towards either diploid, *D. fuchsii* on the Y-axis and *D. incarnata* on the X-axis for 20-23 nt smRNAs target regions found in annotated TEs. Top row shows with coloured symbols transgressive and additive patterns; whereas bottom row shows dominant to either diploid.

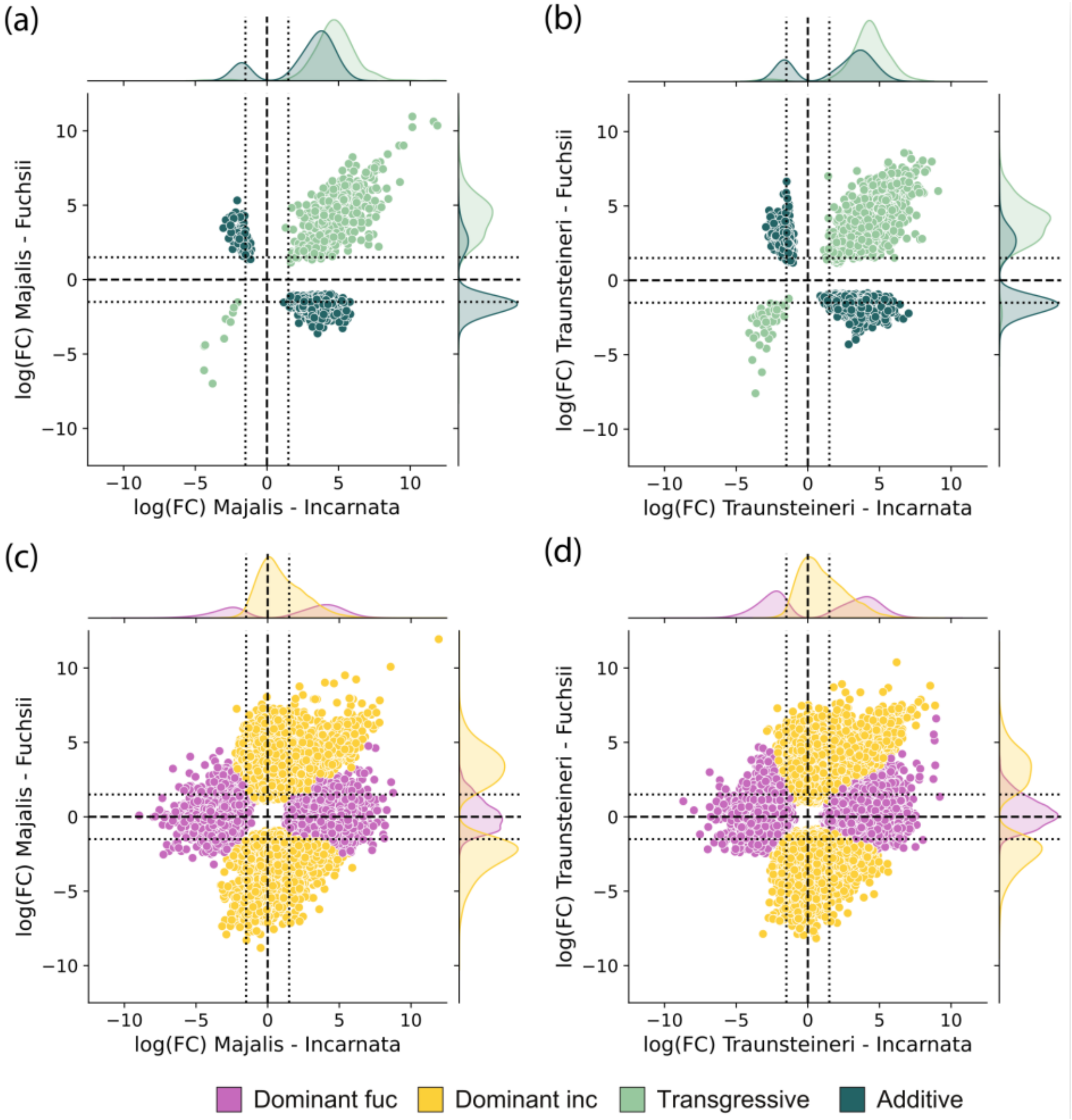

Fig. S10 Patterns of genomic interactions comparing logFC values for *D. majalis* (left) and *D. traunsteineri* (right) towards either diploid, *D. fuchsii* on the Y-axis and *D. incarnata* on the X-axis for 24 nt smRNAs target regions found in annotated TEs. Top row shows with coloured symbols transgressive and additive patterns; whereas bottom row shows dominant to either diploid.

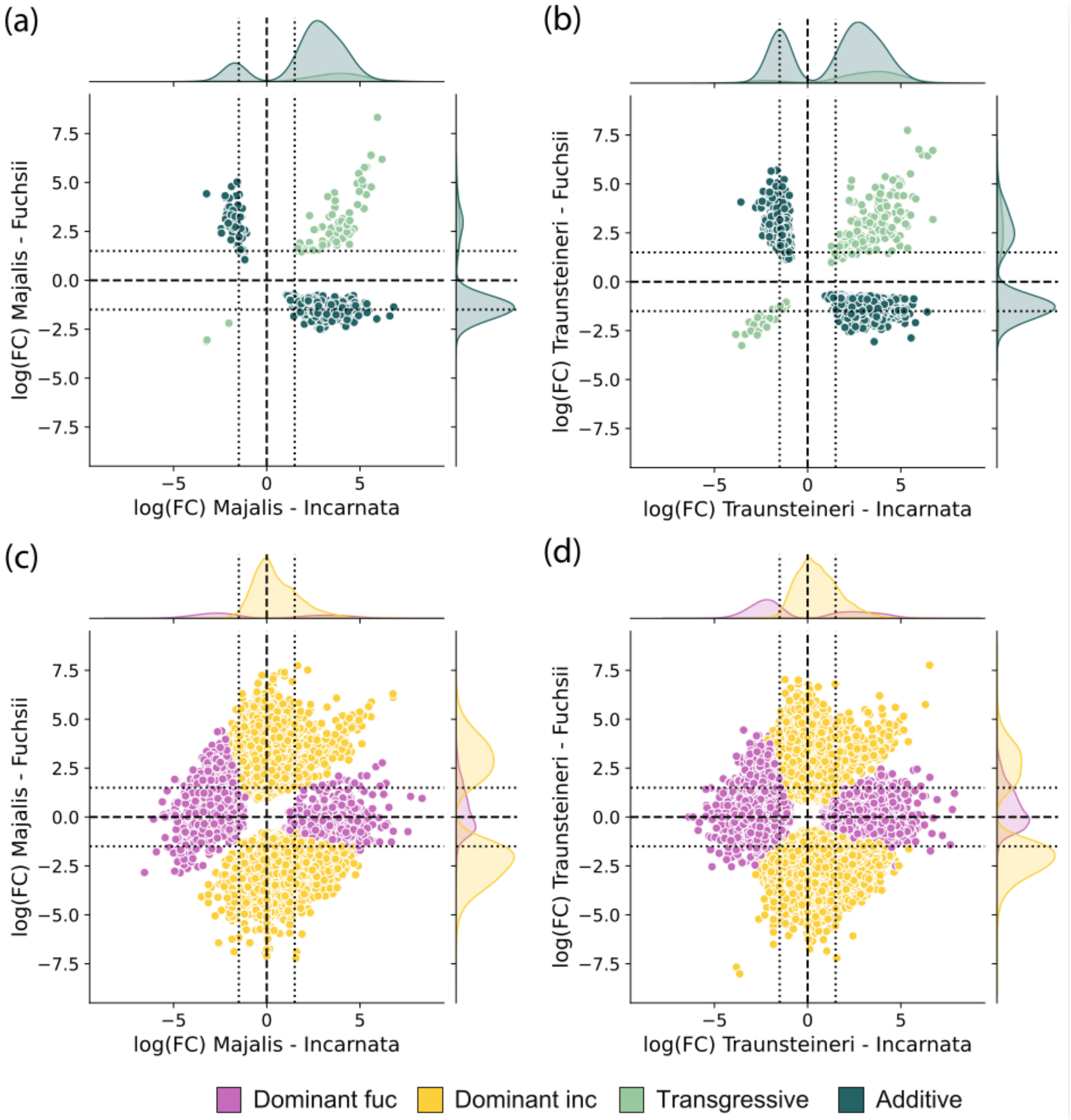

Supplement: Supplementary file 1 — Fig. S1 Distribution of normalized reads counts for different smRNA lengths over genomic regions (promoter/1000 bp upstream, exons, introns and intergenic) in the four tested species. Fig. S2 Number of DT regions in genes and TEs for each comparison between a polyploid (4×) and a diploid (2×) for all three data sets, 20–24 nt, 20–23 nt and 24 nt smRNAs. Fig. S3 Number of DT regions in all datasets 20–24 nt, 20–23 nt and 24 nt smRNAs for each genomic interaction (additive, transgressing or dominant to either diploid). Fig. S4 Patterns of genomic interactions comparing logFC values for D. majalis and D. traunsteineri towards either diploid, D. fuchsii on the y‐axis and D. incarnata on the x‐axis for all target regions (i.e. 20–24 nt smRNAs) found in annotated genes. Fig. S5 Proportions of target regions within each genomic interaction (additive, transgressing, or dominant to either diploid) for each genomic region (1000 bp upstream/promoter, exon, intron). Fig. S6 MA plots showing differences between D. majalis and D. traunsteineri for TEs types grouped together, the central dashed line marks 0 while the dotted lines mark logFC −1.5 and 1.5. Fig. S7 MA plots showing differences between D. majalis and D. traunsteineri for TEs, the central dashed line marks 0 while the dotted lines mark logFC −1.5 and 1.5. Fig. S8 Count of DT regions between D. majalis and D. traunsteineri. Fig. S9 Patterns of genomic interactions comparing logFC values for D. majalis and D. traunsteineri towards either diploid, D. fuchsii on the y‐axis and D. incarnata on the x‐axis for 20–23 nt smRNAs target regions found in annotated TEs. Fig. S10 Patterns of genomic interactions comparing logFC values for D. majalis and D. traunsteineri towards either diploid, D. fuchsii on the y‐axis and D. incarnata on the x‐axis for 24 nt smRNAs target regions found in annotated TEs. [file NPH-250-648-s001.pdf]
